# Supplementary material for: Is there a sex difference in mortality rates in paediatric intensive care units?: a systematic review
Source: Front Pediatr. 2023 Oct 9;11:1225684. doi: 10.3389/fped.2023.1225684 (PMC10591324; doi:10.3389/fped.2023.1225684)
Supplement: Supplementary file 3 [file Datasheet3.pdf]

Appendix 3. Tables of study summaries

Table1. Summary of studies reporting higher female mortality

Study	Design	Age	Population	% Male	F/M OR	Reported	Estimate	Adjustment
Shanmugham 2018[1]	Prospective	0 - 12y	MV	54.62	10.76			
Rowan 2016[2]	Prospective	median 8.7y	BMT	56.31	5.06			
Ali 2016[3]	Retrospective	0 - 18y	Oncology	60	4.9			
Thukral 2006[4]	Prospective	Median 18m	Whole PICU	63.26	3.73			
Coetzee 2014[5]	Retrospective	0 - 7y	Measles	44.83	2.87			
Sayed 2018[6]	Prospective	1 - 15y	Oncology	63.41	2.64			
Patel 2017[7]	Retrospective	0 - 15y	Trauma	57.72	2.61			
Selewski 2012[8]	Retrospective	0 - 10m	ECMO	60.38	2.19			
Lee 2017[9]	Retrospective	0 - 18y	Abuse/Maltreatment	65.35	2.17			
Aroor 2018[10]	Prospective	0 - 18y	Whole PICU	62.31	2.15			
Topjian 2013[11]	Prospective	>1m	Seizures	56	2.03	OR f/m	2.5	Age
Jacobe 2003[12]	Retrospective	<3.81y	BMT	72.5	2.02			
Hui 2012[13]	Retrospective	0 - 18y	RRT	59.46	2			
Hardelid 2018[14]	Retrospective	<16y	Whole PICU	57.73	1.99	OR f/m	1.91	Age, Race/Ethnicity, Time period, High risk conditions, SES
Al-Ayed 2018[15]	Prospective	0 - 14y	RRT	56.25	1.98			

Study	Design	Age	Population	% Male	F/M OR	Reported	Estimate	Adjustment
Parajuli 2020[16]	Retrospective	0 - 12y	Hemophagocytic Lymphohistiocytosis	59.68	1.81			
Koh 2017[17]	Retrospective	<18y	Pneumonia/Resp tract infection	53.16	1.8			
Sinitsky 2015[18]	Retrospective	0 - 16y	MV	56.67	1.79			
de Souza 2016[19]	Prospective	0 - 17y	Sepsis or shock	52.41	1.79	OR f/m	2.25	Variables not reported
Jeschke 2008[20]	Retrospective	0 - 16y	Burns	59.79	1.78			
Hariharan 2011[21]	Prospective	median 5y	Whole PICU	57.67	1.76			
Paret 1999[22]	Retrospective	50d - 16y	ARDS	61.54	1.75			
Bekhit 2014[23]	Retrospective	0 - 168m	Whole PICU	51.22	1.73			
Mitra 2000[24]	Retrospective	<5y	Patients with Diarrhoea	63.33	1.65			
Egbohou 2019[25]	Retrospective	0 - 15y	Whole PICU	64.23	1.64			
Berndtson 2013[26]	Prospective	<18y	Burns	62.65	1.6			
Erdem 2019[27]	Prospective	<18y	ECMO	52.94	1.57			
Li 2015[28]	Retrospective	median 11/12m	Whole PICU	63.31	1.52			
Abebe 2015[29]	Cross-sectional	0 - 14y	Whole PICU	54.71	1.52			
Malhotra 2020[30]	Retrospective	0 - 15y	Whole PICU	61.62	1.52			
Esteban 2015[31]	Retrospective	0 - 18+y	Severe Health Conditions	57.26	1.5			
An 2016[32]	Retrospective	median 6y	BMT	68.97	1.5			

Study	Design	Age	Population	% Male	F/M OR	Reported	Estimate	Adjustment
Khemani 2009[33]	Retrospective	<18y	Sepsis or shock	62.88	1.49			
Odetola 2005[34]	Retrospective	median 19m	Meningitis	57.42	1.46			
Earan 2016[35]	Retrospective	0 - 15y	Whole PICU	63.61	1.41			
Egbohou 2019[36]	Retrospective	0 - 15y	TBI/Head injury	67.03	1.38			
Hsiao 2015[37]	Retrospective	0 - 12y	Pneumococcal disease	39.58	1.33			
Scholefield 2015[38]	Retrospective	0 - 16y	Out of hospital cardiac arrest	58.4	1.33			
Ping 2018[39]	Prospective	median 1.4y	Long stay	51.45	1.32			
Verlaat 2019[40]	Retrospective	<18y	Complex Chronic Conditions	57.72	1.3	OR m/f	0.75	Age, Primary diagnosis, Admission time, Chronic Conditions
Bhaskar 2015[41]	Case	0 - 18y	Sepsis or shock	58.77	1.29			
Choudhary 2020[42]	Prospective	0 - 14y	RRT	62	1.27			
Du 2020[43]	Retrospective	<16y	Whole PICU	58.29	1.27			
Riyuzo 2017[44]	Retrospective	1 - 12y	AKI	61.04	1.24			
Punchak 2018[45]	Retrospective	0 - 15y	Whole PICU	56.82	1.21			
Iro 2019[46]	Retrospective	0 - 17y	Encephalitis	55	1.21			
Balit 2016[47]	Retrospective	<18y	BMT	59.22	1.2	OR f/m	1.2	Unadjusted
Sachdev 2018[48]	Prospective	median 72m	Whole PICU	67.33	1.2			

Study	Design	Age	Population	% Male	F/M OR	Reported	Estimate	Adjustment
Mahdi 2018[49]	Cross-sectional	<15y	Whole PICU	59.09	1.19			
Krishnamurthy 2013[50]	Prospective	0 - 13y	AKI	53.7	1.14			
Patki 2014[51]	Prospective	0 - 16y	Whole PICU	64.36	1.14			
Ferreira 2020[52]	Retrospective	0 - 13y	AKI	56.68	1.14			
Polito 2020[53]	Retrospective	<16y	Whole PICU	57.16	1.14			
Horoz 2019[54]	Prospective	0 - 18y	Intra-abdominal hypertension	54.29	1.11			
Raymakers-Janssen 2019[55]	Retrospective	median 8.9y	BMT	63.24	1.11			
Fraser 2018[56]	Retrospective	0 - 16y	Life-limiting conditions	56.71	1.09	OR f/m	1.09	Age, PIM, Race/Ethnicity, Primary diagnosis, SES, Centre/Unit
Verlaat 2017[57]	Prospective	<18y	Low risk PICU patients	57.5	1.07			
Yadav 2019[58]	Prospective	0 - 14y	ARDS	66.94	1.07			
Epstein 2011[59]	Retrospective	0 - 18y	Whole PICU	55.8	1.06	OR f/m	1.12	Age, PIM, Race/Ethnicity, Insurance type, Primary diagnosis
AbdAllah 2016[60]	Observation	0 - 15y	Whole PICU	51.2	1.06			
AlKadhem 2020[61]	Prospective	0 - 14y	Whole PICU	53.24	1.04			
Klein 2008[62]	Prospective	Mean 82.4m	Whole PICU	51.94	1.03			

Study	Design	Age	Population	% Male	F/M OR	Reported	Estimate	Adjustment
McCrory 2017[63]	Retrospective	<18y	Whole PICU	56	1.03	OR f/m	1.03	Age, PIM, Admission time, Origin of admission, Centre/Unit, Staffing, Trauma
Moynihan 2019[64]	Retrospective	<16y	Whole PICU	57.26	1.02			
Alobaidi 2020[65]	Retrospective	0 - 17y	AKI	56.54	1.01			
Branco 2005[66]	Prospective	mean 33.8m	Sepsis or shock	66.67		OR m/f	0.7	PRISM, Hyperglycaemia
Jaramillo-Bustamante 2012[67]	Prospective	0 - 18y	Sepsis or shock	55		% m/f	18% vs 18.5%	Unadjusted
Ferguson 2012[68]	Prospective	0 - 16y	Arrest	57.92		OR m/f	0.86	Age, PIM, Race/Ethnicity, Time period, Inotropes, Mechanical ventilation

Table 2. Summary of studies reporting equal male and female mortality

Study	Design	Age	Population	% Male	F/M OR	Reported	Estimate	Adjustment
Cairo 2018[69]	Prospective	0 - 18y	ECMO	53.14		OR m/f dx alive	0.98	Age, Race/Ethnicity, Time period, Mechanical ventilation
Tyagi 2018[70]	Prospective	0 - 12y	Whole PICU	56.29	0.98			
Bejersten 1988[71]	Prospective	0 - 15y	Whole PICU	56.05	0.99			
Volakli 2012[72]	Prospective	0 - 14y	Whole PICU	65.33	0.99			
Purcell 2020[73]	Prospective	0 - 18y	Whole PICU	43.81	1	RR f/m	0.97	Age, Inotropes, TBI, qSOFA, Sepsis, Mechanical ventilation, Malaria parasite
Arias 2004[74]	Retrospective	Children	Whole PICU	57.27		OR m/f	1	Age, Admission time, Origin of admission, PRISM, Previous ICU/PICU, Inotropes, Post-op care

F/M OR: This is the female/male OR we calculated from the crude numbers provided, Reported: This is the measure provided by the authors of the study, Adjustment: Any variables used for adjustment for the reported measure, AKI: Acute kidney injury, ARDS: Acute respiratory distress syndrome, Arrest: Cardiorespiratory arrest, BG: Blood glucose, ECMO: Extra-corporeal membrane oxygenation, MOF: Multi organ failure, MV: Mechanical ventilation, RRT: Renal replacement therapy, TBI: Traumatic brain injury, SES: Socio-economic status

Table 3. Summary of studies reporting higher male mortality

Study	Design	Age	Population	% Male	F/M OR	Reported	Estimate	Adjustment
Hon 2016[75]	Retrospective	<12y	Encephalitis	60.87	0.14			
Dursun 2020[76]	Retrospective	<18y	Oncology	43.75	0.18			
Wang 2008[77]	Prospective	0 - 18y	HFOV	60.61	0.19			
Piastra 2019[78]	Retrospective	<14y	Cerebral haemorrhage	56.07	0.29			
Zobel 1991[79]	Retrospective	mean 4.3y	RRT	69.23	0.36			
Branco 2011[80]	Prospective	0 - 16y	MV	47.92	0.4			
Berger 2013[81]	Prospective	<18y	Pertussis infection	51.15	0.49			
Lefevre 2017[82]	Retrospective	0 - 12y	Sepsis or shock	53.52	0.51			
Lombel 2012[83]	Retrospective	median 184m	BMT	66.67	0.53			
Torres 2012[84]	Retrospective	0 - 110m	Influenza	60.56	0.56			
Nyirasafari 2017[85]	Prospective	0 - 15y	Whole PICU	60	0.57	OR f/m	0.63	Nutritional status, diagnosis, modified PRISM score, CPAP, vaso-active drugs
Kaur 2014[86]	Prospective	0 - 14y	Sepsis or shock	60	0.58			
Joffre 2016[87]	Retrospective	0 - 16y	Down's	66.67	0.61			
Muttath 2019[88]	Prospective	0 - 16y	Sepsis or shock	47.08	0.61			
Misirlioglu 2018[89]	Retrospective	mean 5.2y	Whole PICU	53.13	0.63			
Appiah 2018[90]	Retrospective	0 - 13y	Arrest	58.25	0.63			

Study	Design	Age	Population	% Male	F/M OR	Reported	Estimate	Adjustment
Celiny 2007[91]	Retrospective	1m - 15y	Sepsis or shock	66.67	0.67			
Tong 2016[92]	Retrospective	<12y	Paramyxovirus Infection	47.96	0.67			
Cortina 2019[93]	Retrospective	median 5.6y	RRT	52.8	0.69			
Choi 2017[94]	Retrospective	mean 9.5y	AKI	44.72	0.7			
Basnet 2014[95]	Prospective	0 - 16y	Whole PICU	56.56	0.72			
El-Mekkawy 2020[96]	Prospective	0 - 16y	Whole PICU	55.13	0.73			
Ghani 2012[97]	Prospective	median 4.7m	Viral respiratory tract infections	49.14	0.77			
Sik 2019[98]	Retrospective	0 - 18y	RRT	42.86	0.77			
Sial 2019[99]	Observation	1 - 17y	Oncology	61.02	0.8			
Andersson 2019[100]	Retrospective	0 - 18y	MOF	57.14	0.8			
Wong 2019[101]	Retrospective	0 - 157y	Sepsis or shock	44.83	0.81			
Kim 2020[102]	Prospective	median 13y	Oncology	56.36	0.81			
Dewi 2020[103]	Retrospective	0 - 18y	Whole PICU	60.67	0.81			
Chong 2015[104]	Retrospective	<16y	TBI/Head injury	63.64	0.85			
Naghib 2010[105]	Retrospective	Children	Long stay	57.14	0.86			
Patki 2017[106]	Prospective	0 - 16y	Whole PICU	57.86	0.86			
Siddiqui 2018[107]	Retrospective	0 - 16y	Whole PICU	58.68	0.86			
Proulx 1994[108]	Retrospective	<18y	MOF	58.82	0.87			
Lopez 2006[109]	Prospective	0 - 18y	Whole PICU	57.49	0.87			

Study	Design	Age	Population	% Male	F/M OR	Reported	Estimate	Adjustment
Kim 2020[110]	Retrospective	0 - 18y	RBC distribution width reading	51.56	0.87			
Tijssen 2020[111]	Retrospective	<18y	Transported to PICU	58.28	0.87			
Saboktakin 2020[112]	Prospective	0 - 13y	Pneumonia/Resp tract infection	57.92	0.88			
Miklaszewska 2019[113]	Retrospective	mean 9.3y	RRT	58.7	0.89			
Ghuman 2013[114]	Retrospective	2 -7y	Sepsis or shock	52.7	0.9			
Kanwaljeet 2015[115]	Prospective	<18y	Whole PICU	56.74	0.9	OR m/f	1.1	Age, Race/Ethnicity, Insurance type, Sepsis
Rusmawatiningtyas 2016[116]	Prospective	0 - 17y	Sepsis or shock	52.21	0.9			
Selewski 2011[117]	Retrospective	median 19m	RRT	59.29	0.91			
Modem 2014[118]	Retrospective	median 9.5y	RRT	41.05	0.91			
Patel 2017[119]	Retrospective	0 - 18y	Intoxication	44.39	0.93			
Hon 2017[120]	Retrospective	<12y	Whole PICU	55.45	0.93			
Kazantzi 2017[121]	Retrospective	median 60d	Pertussis infection	32.26	0.94			
Li 2019[122]	Prospective	0 - 168m	RBC distribution width reading	61.14	0.97			
Khan 2020[123]	Prospective	0 - 18y	Whole PICU	53.51		OR m/f	1.3	Age, Race/Ethnicity, Primary diagnosis
Memon 2020[124]	Cross-sectional	mean 7y	Oncology	66.67		OR m/f	1.27	Age, Inotropes, Mechanical ventilation

F/M OR: This is the female/male OR we calculated from the crude numbers provided, Reported: This is the measure provided by the authors of the study, Adjustment: Any variables used for adjustment for the reported measure, AKI: Acute kidney injury, ARDS: Acute respiratory distress

Study	Design	Age	Population	% Male	F/M OR	Reported	Estimate	Adjustment

syndrome, Arrest: Cardiorespiratory arrest, BG: Blood glucose, ECMO: Extra-corporeal membrane oxygenation, MOF: Multi organ failure, MV: Mechanical ventilation, RRT: Renal replacement therapy, TBI: Traumatic brain injury, SES: Socio-economic status

References

1. Shanmugham, G., Rajesh, T. V., Francis, C. A. B. & Jayakrishnan, M. P. OUTCOME OF CHILDREN TREATED WITH INVASIVE MECHANICAL VENTILATION IN PICU IN A TERTIARY CARE CENTRE, KERALA. *Journal of Evolution of Medical and Dental Sciences-Jemds* **7**, 2342–2346 (2018).
2. Rowan, C. M. *et al.* Invasive Mechanical Ventilation and Mortality in Pediatric Hematopoietic Stem Cell Transplantation: A Multicenter Study. *Pediatric Critical Care Medicine* **17**, 294–302 (2016).
3. Ali, A. M., Sayed, H. A. & Elzembely, M. M. The Outcome of Critically Ill Pediatric Cancer Patients Admitted to the Pediatric Intensive Care Unit in a Tertiary University Oncology Center in a Developing Country: A 5-Year Experience. *Journal of Pediatric Hematology/Oncology* **38**, 355–359 (2016).
4. Thukral, A., Lodha, R., Irshad, M. & Arora, N. K. Performance of Pediatric Risk of Mortality (PRISM), Pediatric Index of Mortality (PIM), and PIM2 in a pediatric intensive care unit in a developing country. *Pediatric Critical Care Medicine* **7**, 356–361 (2006).
5. Coetzee, S., Morrow, B. M. & Argent, A. C. Measles in a South African paediatric intensive care unit: Again! *Journal of Paediatrics and Child Health* **50**, 379–385 (2014).
6. Sayed, H. A., Ali, A. M. & Elzembely, M. M. Can pediatric risk of mortality score (PRISM III) be used effectively in initial evaluation and follow-up of critically ill cancer patients admitted to pediatric oncology intensive care unit (POICU)? A prospective study, in a tertiary cancer center in Egypt. *Journal of Pediatric Hematology/Oncology* **40**, 382–386 (2018).
7. Patel, N. *et al.* Trauma related admissions to the PICU at Chris Hani Baragwanath Academic Hospital, Johannesburg. *Pediatric Surgery International* **33**, 1013–1018 (2017).
8. Selewski, D. T. *et al.* Fluid overload and fluid removal in pediatric patients on extracorporeal membrane oxygenation requiring continuous renal replacement therapy. *Critical Care Medicine* **40**, 2694–2699 (2012).
9. Lee, E. P. *et al.* Epidemiology and clinical analysis of critical patients with child maltreatment admitted to the intensive care units. *Medicine (United States)* **96**, e7107 (2017).
10. Aroor, S., Kumar, S., Kini, P. & Mundkur, S. Applicability of paediatric index of mortality 2 score to predict outcome in children admitted to paediatric intensive care unit. *Journal of Nepal Paediatric Society* **38**, 149–152 (2018).
11. Topjian, A. A. *et al.* Electrographic Status Epilepticus Is Associated With Mortality and Worse Short-Term Outcome in Critically Ill Children*. *Critical Care Medicine* **41**, 215–223 (2013).
12. Jacobe, S. J., Hassan, A., Veys, P. & Mok, Q. Outcome of children requiring admission to an intensive care unit after bone marrow transplantation. *Critical Care Medicine* **31**, 1299–1305 (2003).

13. Hui, W. F., Chan, W. K. Y. & Lee, K. W. Children on continuous renal replacement therapy: Prognostic factors. *Hong Kong Medical Journal* **18**, 475–481 (2012).
14. Hardelid, P. *et al.* Characteristics and mortality risk of children with life-threatening influenza infection admitted to paediatric intensive care in England 2003–2015. *Respiratory Medicine* **137**, 23–29 (2018).
15. Al-Ayed, T., Siddiqui, N. U. R., Alturki, A. & Aljofan, F. Outcome of continuous renal replacement therapy in critically ill children: A retrospective cohort study. *Annals of Saudi Medicine* **38**, 260–268 (2018).
16. Parajuli, B. *et al.* Hemophagocytic Lymphohistiocytosis in a PICU of a Developing Economy: Clinical Profile, Intensive Care Needs, Outcome, and Predictors of Mortality. *Pediatric critical care medicine : a journal of the Society of Critical Care Medicine and the World Federation of Pediatric Intensive and Critical Care Societies* **22**, e44–e57 (2021).
17. Koh, J. W. J. C. *et al.* Risk factors for mortality in children with pneumonia admitted to the pediatric intensive care unit. *Pediatric Pulmonology* **52**, 1076–1084 (2017).
18. Sinitsky, L., Walls, D., Nadel, S. & Inwald, D. P. Fluid overload at 48 hours is associated with respiratory morbidity but not mortality in a general PICU: retrospective cohort study. *Pediatric critical care medicine* **16**, 205–9 (2015).
19. de Souza, D. C. *et al.* Epidemiology of Sepsis in Children Admitted to PICUs in South America. *Pediatric critical care medicine* **17**, 727–34 (2016).
20. Jeschke, M. G. *et al.* Gender Differences in Pediatric Burn Patients: Does It Make a Difference? *Annals of Surgery* **248**, 126–136 (2008).
21. Hariharan, S., Krishnamurthy, K. & Grannum, D. Validation of Pediatric Index of Mortality-2 scoring system in a pediatric intensive care unit, Barbados. *Journal of tropical pediatrics* **57**, 9–13 (2011).
22. Paret, G. *et al.* Acute respiratory distress syndrome in children: a 10 year experience. *Israel Medical Association Journal* **1**, 149–153 (1999).
23. Bekhit, O. E. S. M., Algameel, A. A. & Eldash, H. H. Application of pediatric index of mortality version 2: score in pediatric intensive care unit in an African developing country. *The Pan African medical journal* **17**, 185 (2014).
24. Mitra, A. K., Rahman, M. M. & Fuchs, G. J. Risk factors and gender differentials for death among children hospitalized with diarrhoea in Bangladesh. *J Health Popul Nutr* **18**, 151–156 (2000).
25. Egbohou, P. *et al.* Pediatric mortality in the multipurpose intensive care unit of Sylvanus Olympio Teaching Hospital (CHU SO) of Lome (Togo). *Pediatric Anesthesia and Critical Care Journal* **7**, 8–12 (2019).
26. Berndtson, A. E., Sen, S., Greenhalgh, D. G. & Palmieri, T. L. Estimating severity of burn in children: Pediatric Risk of Mortality (PRISM) score versus Abbreviated Burn Severity Index (ABSI). *Burns* **39**, 1048–1053 (2013).
27. Erdem, O. *et al.* The Sublingual Microcirculation Throughout Neonatal and Pediatric Extracorporeal Membrane Oxygenation Treatment: Is It Altered by Systemic Extracorporeal Support? *Frontiers in Pediatrics* **7**, (2019).
28. Li, Y. *et al.* U-shaped relationship between early blood glucose and mortality in critically ill children. *BMC Pediatrics* **15**, 88 (2015).
29. Abebe T, Girmay M., Michael G, & Tesfaye M. The epidemiological profile of pediatric patients admitted to the general intensive care unit in an ethiopian university hospital. *International Journal of General Medicine* **8**, 63 (2015).
30. Malhotra, D., Nour, N., El Halik, M. & Zidan, M. Performance and Analysis of Pediatric Index of Mortality 3 Score in a Pediatric ICU in Latifa Hospital, Dubai, UAE. *Dubai Medical Journal* **3**, 19–25 (2020).

31. Esteban E., Bujaldon E., Esparza M., Jordan I., & Esteban M.E. Sex differences in children with severe health conditions: Causes of admission and mortality in a Pediatric Intensive Care Unit. *Am. J. Hum. Biol.* **27**, 613–619 (2015).
32. An, K. *et al.* Prognostic factors and outcome of patients undergoing hematopoietic stem cell transplantation who are admitted to pediatric intensive care unit. *BMC Pediatrics* **16**, 138 (2016).
33. Khemani, R. G. *et al.* Disseminated intravascular coagulation score is associated with mortality for children with shock. *Intensive Care Medicine* **35**, 327 (2009).
34. Odetola, F. O. & Bratton, S. L. Characteristics and immediate outcome of childhood meningitis treated in the pediatric intensive care unit. *Intensive Care Medicine* **31**, 92–97 (2005).
35. Earan, S. K., Dhandapani, L., Arunagirinathan, A. & Kantamneni, S. Clinical Spectrum and Epidemiological Profile of Patients Admitted to Pediatric Intensive Care Unit at a Tertiary Care Centre in South India. *International Journal of Scientific Study* **4**, 187–191 (2016).
36. Egbohou, P. *et al.* Epidemiology of pediatric traumatic brain injury at sylvanus olympio university hospital of lome in Togo. *Anesthesiology Research and Practice* **2019**, 4038319 (2019).
37. Hsiao, H. J. *et al.* Clinical features and outcomes of invasive pneumococcal disease in a pediatric intensive care unit. *Bmc Pediatrics* **15**, (2015).
38. Scholefield, B. R. *et al.* Observational study of children admitted to United Kingdom and Republic of Ireland Paediatric Intensive Care Units after out-of-hospital cardiac arrest. *Resuscitation* **97**, 122–128 (2015).
39. Ping Kirk, A. H. *et al.* Characteristics and Outcomes of Long-Stay Patients in the Pediatric Intensive Care Unit. *Journal of Pediatric Intensive Care* **7**, 1–6 (2018).
40. Verlaat, C. W. *et al.* Retrospective cohort study on factors associated with mortality in high-risk pediatric critical care patients in the Netherlands. *BMC pediatrics* **19**, 274 (2019).
41. Bhaskar, P., Dhar, A. V., Thompson, M., Quigley, R. & Modem, V. Early fluid accumulation in children with shock and ICU mortality: a matched case-control study. *Intensive Care Medicine* **41**, 1445–1453 (2015).
42. Choudhary, P., Kumar, V., Saha, A. & Thakur, A. Peritoneal dialysis in critically ill children in resource-limited setting: A prospective cohort study. *Peritoneal Dialysis International* (2020) doi:10.1177/0896860820975897.
43. Du, Y. *et al.* Glycemic Variability: An Independent Predictor of Mortality and the Impact of Age in Pediatric Intensive Care Unit. *Frontiers in Pediatrics* **8**, 403 (2020).
44. Riyuzo, M. C., Silveira, L. V. D., Macedo, C. S. & Fioretto, J. R. Predictive factors of mortality in pediatric patients with acute renal injury associated with sepsis. *Jornal De Pediatria* **93**, 28–34 (2016).
45. Punchak, M. *et al.* Epidemiology of Disease and Mortality From a PICU in Mozambique. *Pediatric critical care medicine* **19**, e603–e610 (2018).
46. Iro, M. A., Sadarangani, M., Nickless, A., Kelly, D. F. & Pollard, A. J. A Population-based Observational Study of Childhood Encephalitis in Children Admitted to Pediatric Intensive Care Units in England and Wales. *The Pediatric infectious disease journal* **38**, 673–677 (2019).
47. Balit, C. R. *et al.* Pediatric hematopoietic stem cell transplant and intensive care: Have things changed? *Pediatric Critical Care Medicine* **17**, e109–e116 (2016).
48. Sachdev, A. *et al.* Outcome Prediction Value of Red Cell Distribution Width in Critically-ill Children. *Indian Pediatrics* **55**, 414–416 (2018).

49. Mahdi, A. H. Profile of patients admitted to pediatric intensive care unit, babylon teaching hospital for gynecology and children. *Research Journal of Pharmacy and Technology* **11**, 2283–2288 (2018).
50. Krishnamurthy, S. *et al.* Clinical profile of acute kidney injury in a pediatric intensive care unit from Southern India: A prospective observational study. *Indian Journal of Critical Care Medicine* **17**, 207–213 (2013).
51. Patki, V. K. & Chougule, S. B. Hyperglycemia in critically ill children. *Indian Journal of Critical Care Medicine* **18**, 8–13 (2014).
52. Ferreira, M. C. D. R. & Lima, E. Q. Impact of the development of acute kidney injury on patients admitted to the pediatric intensive care unit. *Jornal de Pediatria* **96**, 576–581 (2020).
53. Polito, A., Giacobino, C., Combescure, C., Levy-Jamet, Y. & Rimensberger, P. Overall and subgroup specific performance of the pediatric index of mortality 2 score in Switzerland: a national multicenter study. *European Journal of Pediatrics* **179**, 1515–1521 (2020).
54. Horoz, O. O. *et al.* The relationship of abdominal perfusion pressure with mortality in critically ill pediatric patients. *Journal of Pediatric Surgery* **54**, 1731–1735 (2019).
55. Raymakers-Janssen, P. A. M. A. *et al.* Epidemiology and Outcome of Critically Ill Pediatric Cancer and Hematopoietic Stem Cell Transplant Patients Requiring Continuous Renal Replacement Therapy: A Retrospective Nationwide Cohort Study. *Critical care medicine* **47**, e893–e901 (2019).
56. Fraser, L. K. & Parslow, R. Children with life-limiting conditions in paediatric intensive care units: A national cohort, data linkage study. *Archives of Disease in Childhood* **103**, 540–547 (2018).
57. Verlaat, C. W. *et al.* Factors Associated with Mortality in Low-Risk Pediatric Critical Care Patients in the Netherlands. *Pediatric Critical Care Medicine* **18**, e155–e161 (2017).
58. Yadav, B., Bansal, A. & Jayashree, M. Clinical Profile and Predictors of Outcome of Pediatric Acute Respiratory Distress Syndrome in a PICU: A Prospective Observational Study. *Pediatric Critical Care Medicine* **20**, e263–e273 (2019).
59. Epstein, D. *et al.* Race/Ethnicity is not associated with mortality in the PICU. *Pediatrics* **127**, e588-597 (2011).
60. AbdAllah, N. B., Zeitoun, A. E. & Fattah, M. Adherence to standard admission and discharge criteria and its association with outcome of pediatric intensive care unit cases in Al-Ahrar Hospital Zagazig. *Egyptian Pediatric Association Gazette* **64**, 111–119 (2016).
61. AlKadhem, S. M. *et al.* The Association Between Admission Sources and Outcomes at a Pediatric Intensive Care Unit in Al-Ahsa, Saudi Arabia: A Retrospective Cohort Study. *Cureus* **12**, (2020).
62. Klein, G. W., Hojsak, J. M., Schmeidler, J. & Rapaport, R. Hyperglycemia and Outcome in the Pediatric Intensive Care Unit. *Journal of Pediatrics* **153**, 379 (2008).
63. McCrory, M. C. *et al.* Time of admission to the PICU and mortality. *Pediatric Critical Care Medicine* **18**, 915–923 (2017).
64. Moynihan, K. M. *et al.* Epidemiology of childhood death in Australian and New Zealand intensive care units. *Intensive Care Medicine* **45**, 1262–1271 (2019).
65. Alobaidi, R., Morgan, C., Goldstein, S. L. & Bagshaw, S. M. Population-Based Epidemiology and Outcomes of Acute Kidney Injury in Critically Ill Children. *Pediatric critical care medicine* **21**, 82–91 (2020).
66. Branco, R. G. *et al.* Glucose level and risk of mortality in pediatric septic shock. *Pediatric Critical Care Medicine* **6**, 470–472 (2005).

67. Jaramillo-Bustamante, J. C., Marín-Agudelo, A., Fernández-Laverde, M. & Bareño-Silva, J. Epidemiology of sepsis in pediatric intensive care units: First Colombian multicenter study. *Pediatr Crit Care Med* **13**, 501–508 (2012).
68. Ferguson Lee P., Durward Andrew, & Tibby Shane M. Relationship Between Arterial Partial Oxygen Pressure After Resuscitation From Cardiac Arrest and Mortality in Children. *Circulation* **126**, 335–342 (2012).
69. Cairo, S. B. *et al.* Comparing Percutaneous to Open Access for Extracorporeal Membrane Oxygenation in Pediatric Respiratory Failure. *Pediatric Critical Care Medicine* **19**, 981–991 (2018).
70. Tyagi, P., Tullu, M. S. & Agrawal, M. Comparison of Pediatric Risk of Mortality III, Pediatric Index of Mortality 2, and Pediatric Index of Mortality 3 in Predicting Mortality in a Pediatric Intensive Care Unit. *Journal of Pediatric Intensive Care* **7**, 201–206 (2018).
71. Bejersten, A. & Olsson, G. L. The outcome of mechanically ventilated infants and children. *International journal of clinical monitoring and computing* **5**, 91–6 (1988).
72. Volakli, E. A. *et al.* Short-term and long-term mortality following pediatric intensive care. *Pediatrics International* **54**, 248–255 (2012).
73. Purcell, L. N. *et al.* Outcomes Following Intensive Care Unit Admission in a Pediatric Cohort in Malawi. *Journal of tropical pediatrics* **66**, 621–629 (2020).
74. Arias, Y., Taylor, D. S. & Marcin, J. P. Association between evening admissions and higher mortality rates in the pediatric intensive care unit. *Pediatrics* **113**, e530-534 (2004).
75. Hon, K. L. E. *et al.* Outcome of Encephalitis in Pediatric Intensive Care Unit. *Indian Journal of Pediatrics* **83**, 1098–1103 (2016).
76. Dursun, A., Ozsoylu, S. & Akyildiz, B. N. Outcomes and prognostic factors for pediatric cancer patients admitted to an intensive care unit in a university hospital. *Turkish Journal of Pediatrics* **62**, 252–258 (2020).
77. Wang, C. C., Wu, W. L., Wu, E. T., Chou, H. C. & Lu, F. L. High frequency oscillatory ventilation in children: Experience of a Medical Center in Taiwan. *Journal of the Formosan Medical Association* **107**, 311–315 (2008).
78. Piastra, M. *et al.* Clinical Outcomes and Prognostic Factors for Spontaneous Intracerebral Hemorrhage in Pediatric ICU: A 12-Year Experience. *Journal of Intensive Care Medicine* **34**, 1003–1009 (2019).
79. Zobel, G., Ring, E., Kuttnig, M. & Grubbauer, H. M. Five years experience with continuous extracorporeal renal support in paediatric intensive care. *Intensive Care Medicine* **17**, 315–319 (1991).
80. Branco, R. G. *et al.* Prospective operationalization and feasibility of a glycemic control protocol in critically ill children. *Pediatric Critical Care Medicine* **12**, 265–270 (2011).
81. Berger, J. T. *et al.* Critical pertussis illness in children: a multicenter prospective cohort study. *Pediatric critical care medicine* **14**, 356 (2013).
82. Lefèvre, N. *et al.* Sex Differences in Inflammatory Response and Acid-Base Balance in Prepubertal Children with Severe Sepsis. *Shock* **47**, 422–428 (2017).
83. Lombel, R. M. *et al.* Implications of different fluid overload definitions in pediatric stem cell transplant patients requiring continuous renal replacement therapy. *Intensive Care Medicine* **38**, 663–669 (2012).
84. Torres, S. F. *et al.* High mortality in patients with influenza A pH1N1 2009 admitted to a pediatric intensive care unit: a predictive model of mortality. *Pediatric critical care medicine* **13**, e78-83 (2012).

85. Nyirasafari, R. *et al.* Predictors of mortality in a paediatric intensive care unit in Kigali, Rwanda. *Paediatrics and International Child Health* **37**, 109–115 (2017).
86. Kaur, G., Vinayak, N., Mittal, K., Kaushik, J. S. & Aamir, M. Clinical outcome and predictors of mortality in children with sepsis, severe sepsis, and septic shock from Rohtak, Haryana: A prospective observational study. *Indian Journal of Critical Care Medicine* **18**, 437–441 (2014).
87. Joffre, C., Lesage, F., Bustarret, O., Hubert, P. & Oualha, M. Children with Down syndrome: Clinical course and mortality-associated factors in a French medical paediatric intensive care unit. *Journal of Paediatrics and Child Health* **52**, 595–599 (2016).
88. Muttath, A., Annayappa Venkatesh, L., Jose, J., Vasudevan, A. & Ghosh, S. Adverse Outcomes due to Aggressive Fluid Resuscitation in Children: A Prospective Observational Study. *Journal of Pediatric Intensive Care* **8**, 64–70 (2019).
89. Misirlioglu, M., Bekdas, M. & Kabakus, N. Platelet-lymphocyte ratio in predicting mortality of patients in pediatric intensive care unit. *Journal of Clinical and Analytical Medicine* **9**, 488–492 (2018).
90. Appiah, J., Salie, S., Argent, A. & Morrow, B. Characteristics, course and outcomes of children admitted to a paediatric intensive care unit after cardiac arrest. *Southern African Journal of Critical Care* **34**, 58–64 (2018).
91. Pedro Celiny Ramos, G. *et al.* Ferritin levels in children with severe sepsis and septic shock. *Acta paediatrica (Oslo, Norway : 1992)* **96**, 1829 (2007).
92. Tong, S. W. A. *et al.* Paramyxovirus infection: Mortality and morbidity in a pediatric intensive care unit. *Journal of Tropical Pediatrics* **62**, 352–360 (2016).
93. Cortina, G. *et al.* Mortality of Critically Ill Children Requiring Continuous Renal Replacement Therapy: Effect of Fluid Overload, Underlying Disease, and Timing of Initiation. *Pediatric critical care medicine* **20**, 314–322 (2019).
94. Choi, S. J., Ha, E.-J., Jhang, W. K. & Park, S. J. Factors Associated With Mortality in Continuous Renal Replacement Therapy for Pediatric Patients With Acute Kidney Injury. *Pediatric critical care medicine* **18**, e56–e61 (2017).
95. Basnet, S. *et al.* Development of a PICU in Nepal: The experience of the first year. *Pediatric Critical Care Medicine* **15**, e314–e320 (2014).
96. El-Mekkawy, M. S., Ellahony, D. M., Khalifa, K. A. E. & Abd Elsattar, E. S. Plasma lactate can improve the accuracy of the Pediatric Sequential Organ Failure Assessment Score for prediction of mortality in critically ill children: A pilot study. *Archives de Pediatrie* **27**, 206–211 (2020).
97. Ghani, A. S. A., Morrow, B. M., Hardie, D. R. & Argent, A. C. An investigation into the prevalence and outcome of patients admitted to a pediatric intensive care unit with viral respiratory tract infections in Cape Town, South Africa. *Pediatric Critical Care Medicine* **13**, e275–e281 (2012).
98. Sik, G., Demirbuga, A., Gunhar, S., Nisli, K. & Citak, A. Clinical Features and Indications Associated with Mortality in Continuous Renal Replacement Therapy for Pediatric Patients. *Indian Journal of Pediatrics* **86**, 360–364 (2019).
99. Sial, G. Z. K. & Khan, S. J. Pediatric Cancer Outcomes in an Intensive Care Unit in Pakistan. *Journal of Global Oncology* **5**, (2019).
100. Andersson, A., Norberg, A., Broman, L. M., Martensson, J. & Flaring, U. Fluid balance after continuous renal replacement therapy initiation and outcome in paediatric multiple organ failure. *Acta Anaesthesiologica Scandinavica* **63**, 1028–1036 (2019).
101. Wong, J. J. *et al.* Positive Fluid Balance is Associated with Poor Clinical Outcomes in Paediatric Severe Sepsis and Septic Shock. *Annals of the Academy of Medicine, Singapore* **48**, 290–297 (2019).
102. Kim, K. *et al.* Prognostic Factors of ICU Mortality in Pediatric Oncology Patients With Pulmonary Complications. *Journal of Pediatric Hematology Oncology* **42**, 266–270 (2020).

103. Dewi, G. *et al.* Pediatric Index of Mortality 3 score as a predictor for the outcomes of critically ill patients. *Paediatrica Indonesiana* **60**, 328–333 (2020).
104. Chong, S. L. *et al.* Early hyperglycemia in pediatric traumatic brain injury predicts for mortality, prolonged duration of mechanical ventilation, and intensive care stay. *International Journal of Endocrinology* **2015**, 719476 (2015).
105. Naghib, S., van der Starre, C., Gischler, S. J., Joosten, K. F. M. & Tibboel, D. Mortality in very long-stay pediatric intensive care unit patients and incidence of withdrawal of treatment. *Intensive Care Med* **36**, 131–136 (2010).
106. Patki, V. K., Antin, J. V. & Khare, S. H. Persistent Hyperlactatemia as the Predictor of Poor Outcome in Critically Ill Children: A Single-Center, Prospective, Observational Cohort Study. *Journal of Pediatric Intensive Care* **6**, 152–158 (2017).
107. Siddiqui, I., Jafri, L., Abbas, Q., Raheem, A. & Ul Haque, A. Relationship of Serum Procalcitonin, C-reactive Protein, and Lactic Acid to Organ Failure and Outcome in Critically III Pediatric Population. *Indian Journal of Critical Care Medicine* **22**, 91–95 (2018).
108. Proulx, F., Gauthier, M., Nadeau, D., Lacroix, J. & Farrell, C. A. Timing and predictors of death in pediatric patients with multiple organ system failure. *Critical Care Medicine* **22**, 1025–1031 (1994).
109. Lopez, A. M. *et al.* Variation in pediatric intensive care therapies and outcomes by race, gender, and insurance status*. *Pediatric Critical Care Medicine* **7**, 2–6 (2006).
110. Kim, D., Ha, E. J., Park, S. J. & Jhang, W. K. Evaluation of the usefulness of red blood cell distribution width in critically ill pediatric patients. *Medicine* **99**, (2020).
111. Tijssen, J. A. *et al.* Paediatric health care access in community health centres is associated with survival for critically ill children who undergo inter-facility transport: A province-wide observational study. *Paediatrics and Child Health (Canada)* **25**, 308–316 (2020).
112. Saboktakin, L. Pneumonia-Associated Hypocalcaemia as a Poor Prognostic Factor in the Clinical Outcomes of Infant and Pediatric Intensive Care Unit Patients. *Crescent Journal of Medical and Biological Sciences* **7**, 533–538 (2020).
113. Miklaszewska, M. *et al.* Factors affecting mortality in children requiring continuous renal replacement therapy in pediatric intensive care unit. *Advances in Clinical and Experimental Medicine* **28**, 615–623 (2019).
114. Ghuman, A. K., Newth, C. J. L. & Khemani, R. G. Impact of Gender on Sepsis Mortality and Severity of Illness for Prepubertal and Postpubertal Children. *The Journal of Pediatrics* **163**, 835-840.e1 (2013).
115. Kanwaljeet, J. S. A., Robert, J. S., Kimberley, G., Samir, H. S. & Paul, D. J. Pediatric intensive care unit mortality among Latino children before and after a multilevel health care delivery intervention. *JAMA pediatrics* **169**, 383 (2015).
116. Rusmawatiningtyas, D. Mortality rates in pediatric septic shock. *Paediatrica Indonesiana* **56**, 304–310 (2016).
117. Selewski, D. T. *et al.* Weight-based determination of fluid overload status and mortality in pediatric intensive care unit patients requiring continuous renal replacement therapy. *Intensive care medicine* **37**, 1166–73 (2011).
118. Modem, V., Thompson, M., Gollhofer, D., Dhar, A. V. & Quigley, R. Timing of continuous renal replacement therapy and mortality in critically ill children. *Critical Care Medicine* **42**, 943–953 (2014).
119. Patel, M. M. *et al.* Analysis of Interventions Required in 12,021 Children with Acute Intoxications Admitted to PICUs. *Pediatric Critical Care Medicine* **18**, e281–e289 (2017).

120. Hon, K. L. *et al.* Mortality, length of stay, bloodstream and respiratory viral infections in a pediatric intensive care unit. *Journal of Critical Care* **38**, 57–61 (2017).
121. Kazantzi, M. S. *et al.* Characteristics of Bordetella pertussis infection among infantsand children admitted to paediatric intensive care units in Greece: A multicentre, 11-year study. *Journal of Paediatrics and Child Health* **53**, 257–262 (2017).
122. Li, G. *et al.* Usefulness of RBC distribution width and C-reactive protein to predict mortality in pediatric non-cardiac critical illness. *The American journal of emergency medicine* **37**, 2143–2150 (2019).
123. Khan, A. H. *et al.* Pediatric Mortality and Acute Kidney Injury Are Associated with Chloride Abnormalities in Intensive Care Units in the United States: A Multicenter Observational Study. *Journal of Pediatric Intensive Care* (2020) doi:10.1055/s-0040-1719172.
124. Memon, U. A. *et al.* Clinical profile and outcome of carbapenem-resistant gram negative bacteremia in children with cancer in pediatric intensive care unit of a resource-limited country. *Pediatric Hematology Oncology Journal* **5**, 65–68 (2020).
