# Supplementary material for: Is there a sex difference in mortality rates in paediatric intensive care units?: a systematic review
Source: Front Pediatr. 2023 Oct 9;11:1225684. doi: 10.3389/fped.2023.1225684 (PMC10591324; doi:10.3389/fped.2023.1225684)

Appendix 4 (Additional plots for some of the reported sub-populations)

Female/male mortality for PICU patients admitted with sepsis

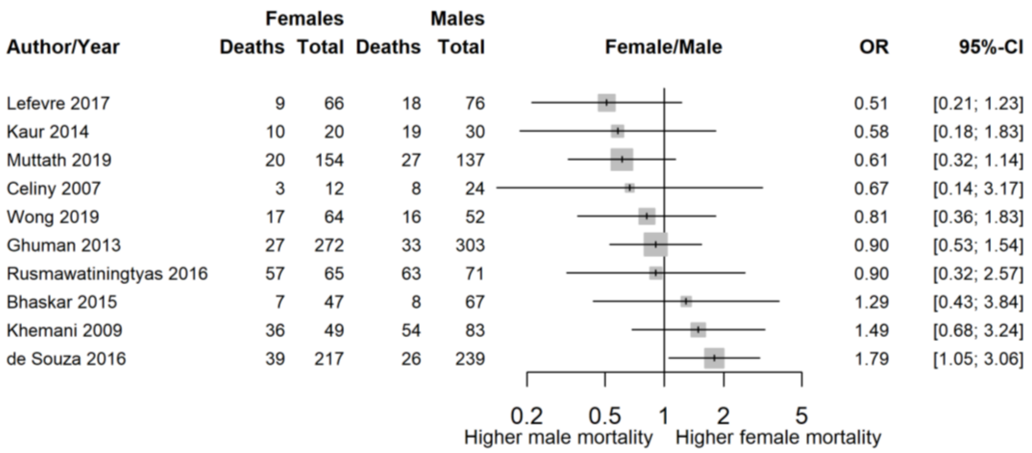

Female/male mortality for PICU patients admitted with renal replacement therapy

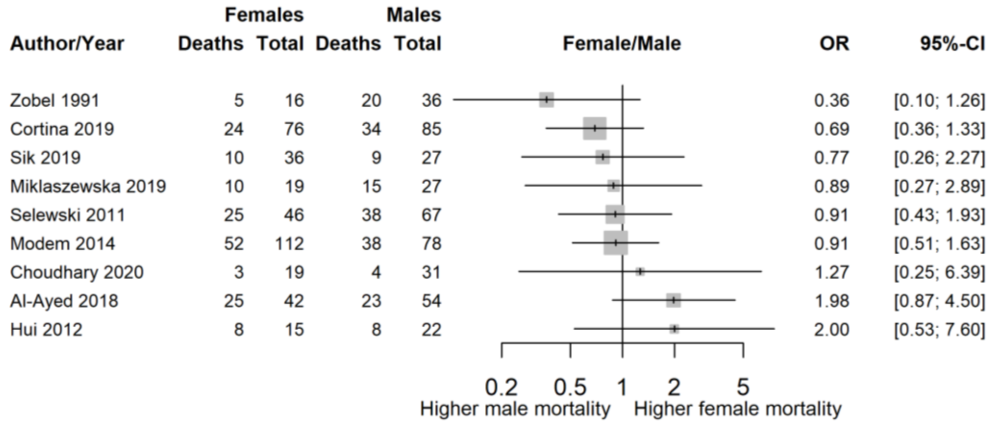

Female/male mortality for PICU patients admitted with bone marrow transplant

Author/Year	Females Deaths	Females Total	Males Deaths	Males Total	OR	95%-CI
Lombel 2012	2	7	6	14	0.53	[0.08; 3.76]
Raymakers-Janssen 2019	14	25	23	43	1.11	[0.41; 2.98]
An 2016	3	9	5	20	1.50	[0.27; 8.34]
Jacobe 2003	9	11	20	29	2.02	[0.36; 11.34]
Rowan 2016	78	97	56	125	5.06	[2.74; 9.34]

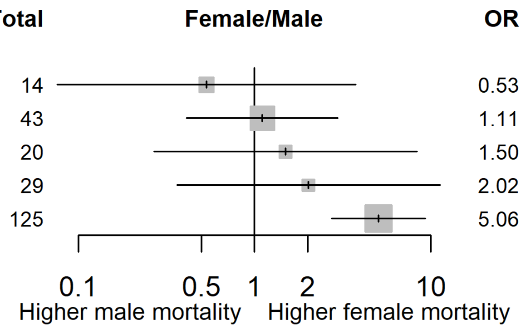

Female/male mortality for PICU patients admitted with oncological conditions

Author/Year	Females Deaths	Females Total	Males Deaths	Males Total	OR	95%-CI
Dursun 2020	17	27	19	21	0.18	[0.03; 0.93]
Sial 2019	9	23	16	36	0.80	[0.28; 2.33]
Kim 2020	27	48	38	62	0.81	[0.38; 1.75]
Sayed 2018	28	45	30	78	2.64	[1.24; 5.61]
Ali 2016	136	220	82	330	4.90	[3.38; 7.08]

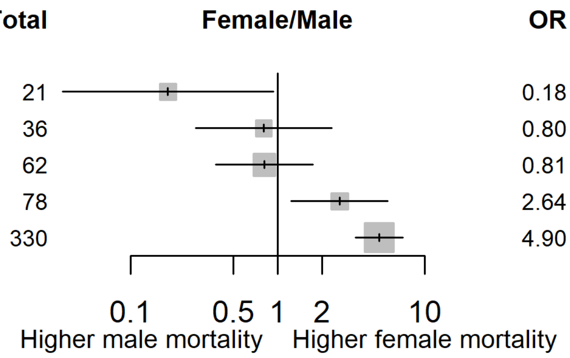

Female/male mortality for PICU patients admitted with acute kidney injury

Author/Year	Females Deaths	Females Total	Males Deaths	Males Total	OR	95%-CI
Choi 2017	25	68	25	55	0.70	[0.34; 1.44]
Alobaidi 2020	14	442	18	575	1.01	[0.50; 2.06]
Krishnamurthy 2013	12	25	13	29	1.14	[0.39; 3.32]
Ferreira 2020	44	188	52	246	1.14	[0.72; 1.80]
Riyuzo 2017	11	30	15	47	1.24	[0.47; 3.24]

Female/Male

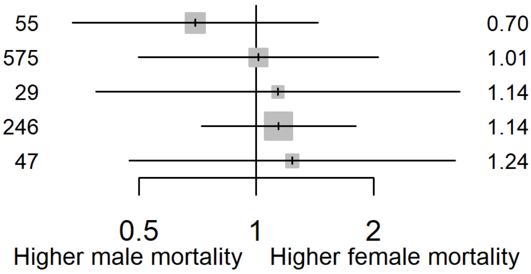

Female/male mortality for PICU patients on mechanical ventilation

Author/Year	Females Deaths	Females Total	Males Deaths	Males Total	OR	95%-CI
Branco 2011	8	75	16	69	0.40	[0.16; 0.99]
Sinitsky 2015	30	276	23	361	1.79	[1.02; 3.16]
Shanmugham 2018	52	59	29	71	10.76	[4.29; 27.00]

Female/Male

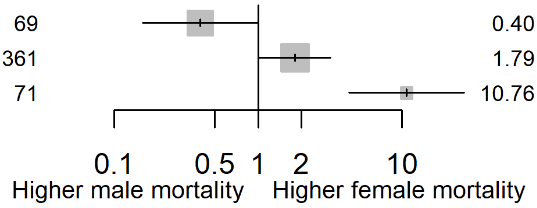

Supplement: Supplementary file 4 [file Datasheet4.pdf]
